# Experiences and Expectations of Ethnic Minorities and Majorities towards Community Pharmacy Medicines-Related Services in Estonia

**DOI:** 10.3390/ijerph19084755

**Published:** 2022-04-14

**Authors:** Kristiina Sepp, Daisy Volmer

**Affiliations:** Institute of Pharmacy, Faculty of Medicine, University of Tartu, 50411 Tartu, Estonia; daisy.volmer@ut.ee

**Keywords:** community pharmacy, medicines-related services, pharmacy customers, experiences, expectations, ethnicity, Russians, Estonia

## Abstract

The unique professional competence of pharmacists can support the safe and effective use of medicines by patients. Additionally, it is important to acknowledge and incorporate the needs of patients with various cultural and social backgrounds. The objective of this study was to assess and compare the experiences and expectations of Russian- and Estonian-speaking pharmacy customers about medicines-related services in Estonian community pharmacies. Cross-sectional study among pharmacy customers was conducted in Estonia 2018–2020. For data analysis, an Independent *t*-Test was used to compare experiences and expectations of respondents towards medicines-related services. The study involved 552 pharmacy customers: 58.5% (*n* = 323) Estonians and 41.5% (*n* = 229) Russians. The majority of the total sample (78.3%) considered the pharmacist competent to help. Medicines-related concerns were more common among Russians (*p* = 0.037), however, they sought less contact to consult a pharmacist than Estonians (*p* < 0.001). Furthermore, expectations about different medicines-related services in the future were higher among Estonians than among Russians (*p* < 0.001). Community pharmacists in Estonia should focus more on person-centered care to better meet the expectations and needs of different ethnic groups about medicines-related services. It is also important to reduce the language barrier and to increase the recognition of cultural traditions by health professionals.

## 1. Introduction

The socio-cultural changes that have taken place in society in recent years also affect people’s behavior and expectations regarding healthcare. New digital solutions and accessibility to services—a mouse click away—engage and empower patients to optimize their own healthcare experience. It also significantly affects and changes the nature and content of patient–healthcare provider contact and there is a clear shift from the concept of universal healthcare to personal counseling of the patient, whereas the number of healthcare providers involved in a patient’s care multiplies across a lifetime [1,2].

More attention has also been paid to the social and cultural determinants in a patient’s health, such as socio-economic-status, race/ethnicity, gender, immigration status and community characteristics [3,4]. Several previous studies have shown that ethnic majorities have a more positive health profile than minorities and that the difference is particularly marked among the elderly [5,6]. Moreover, the assessment of social and cultural determinants of health is complex and often requires consideration of the ethnicity, geographical location, genetic background and exposure to the environment of a particular population group [1,7,8].

The impact of ethnic factors on health, healthcare use and satisfaction is more described in the example of ethnic minorities in the United States [7,8]. There are few studies on the health behavior of ethnic Russians and other primary Russian-speaking communities in the Baltic countries [9,10]. Previous study among older adults in Russia emphasized that health-related habits and social perceptions (e.g., drinking, smoking, poor diet, lack of recreational exercise and a passive approach to health) reflect a communist cultural heritage. The role of the centralized and free state-owned healthcare system is still overestimated, leading to a reduction in responsibility for personal health and lifestyle [11]. Similar problems are described in articles about Russian immigrants in European countries and among the Russian-speaking population in Estonia [12,13]. Russians have stronger trust in institutional measures related to doctors and biomedicine than Estonians and have less willingness to deal with their health individually, whereas younger Estonians see the importance of their own role in health-related topics [14].

Ethnic minorities tend to receive lower quality of care and experience greater morbidity and mortality from various chronic diseases in comparison to non-minorities [14,15]. In the patient–healthcare provider contact, both behave more passive, being less verbally expressive and assertive [16]. Ethnic minorities, who have limited knowledge of the local language may feel embarrassed, further hindering their ability to communicate and to build a solid relationship with healthcare professionals [17]. Implementation of programs reducing language barriers can improve effective involvement of minorities in their own care and ultimately improve the health of the under-served minority population [18].

If we look at pharmacy service as a part of the healthcare service, then, similarly to the above, low satisfaction with the quality of the service has been identified more among the minorities [19,20]. Therefore, it is essential that pharmacists adjust to the needs of patients to deliver the best possible care, considering gender, age and social background, including cultural context and linguistic aspects (spoken or written language barriers) of the patient. The latter acts as a constraint or facilitator of access to information and care [21,22,23]. Contemporary healthcare must be flexible and well-designed, features that cannot often be attributed to traditional healthcare [1]. Greater patient involvement must be ensured in order to achieve improved health outcomes, better patient care and lower costs [24].

The aim of this study was to assess and compare the experiences and expectations of Russian- and Estonian-speaking pharmacy customers regarding medicines-related services in community pharmacies in Estonia.

## 2. Materials and Methods

### 2.1. Study Context

Estonia (population 1.3 million) is a country in the Baltic region of Northern Europe bordered to the north by the Gulf of Finland, to the west by the Baltic Sea, to the south by Latvia and to the east by Lake Peipus and Russia [25,26]. A total of 1 million ethnic Russians live in the Baltics, 300,000 (25% of the population) in Estonia. Only about a third of the Russian speakers also speak Estonian and have Estonian citizenship. The Russian community in Estonia is, to some extent, isolated, as a number of Russians are still connected to their historic homeland Russia [27,28,29].

Estonia restored independence after 50 years of Soviet occupation in 1991 and is known as one of the most digitally advanced societies in the world [30]. After the restoration of independence, a new state had to be built in Estonia, including the healthcare system and pharmaceutical sector [31,32]. Healthcare is based on solidarity financing, whereas the central role is performed by a general practitioner (GP), acting as the first level of contact to the patient [33]. Nevertheless, only 27% of Estonians receive an appointment at the GP on the same day and 25% in more than five days [34]. This problem has been exacerbated by the COVID-19 pandemic—GPs were overburdened and provision of healthcare service was partially impeded at the beginning of the pandemic [35].

Thus, pharmacies have a very important role to play in accessing primary healthcare. Most of the people living in Estonia (94%) visit a community pharmacy at least once in half a year [34]. Community pharmacies are well accessible: for 99% of Estonians, it is 15 min by car [36]. Additionally, pharmacy service is provided over distance by five online pharmacies [37]. On the other hand, community pharmacy service is not defined as a healthcare service and integration into primary healthcare is poor [32,38]. Pharmacy service in Estonia has focused on traditional services such as dispensing and compounding of medicines and counseling on medication use [39]. The development of Community Pharmacy Service Quality Guidelines has initiated a development and standardization of pharmacy services in order to improve the quality aspects of service provision, but also to expand the role of pharmacists in the primary healthcare system [40].

### 2.2. Study Design and Sample

In 2018–2020, the cross-sectional survey was conducted among pharmacy customers at community pharmacies in four different regions of Estonia. The two study regions are inhabited mainly by Estonians; the third, a capital area, is more inhabited with members of national minorities, and the fourth study area, which is next to the Russian border, has a very high proportion of the Russian-speaking population (95%) [41]. A convenience sampling was used and customers purchasing prescription or non-prescription medicines at a community pharmacy were invited to the study. Participation was voluntary and anonymous. The number of pharmacy customers who declined to complete the survey was not recorded. Prior to completing the questionnaires, customers gave informed consent to participate in the study. An approval from the Ethics Committee of the University of Tartu (no. 284/T-1) was received for this study.

### 2.3. Study Instrument

The questionnaire was developed based on a survey instrument which originated in England [42]. To reach the Russian-speaking population, the largest minority in Estonia, forward and backward translation of the study instrument was organized from Estonian into Russian by the native Russian speakers with good knowledge of Estonian. The content of the study instrument was pre-tested among a random sample of pharmacy customers (n = 14; 8 Estonians and 6 Russians), who assessed the clarity and comprehensibility of the instrument. As a result, the questionnaire was modified: the structure and wording were changed for some questions and a question on monthly income was added. No further validation steps, including statistical measures for the survey instrument, were performed due to the exploratory nature of the study.

The questionnaire consisted of 16 items and demographics (gender, age, education, employment status, place of residence, language and income in one month) of the respondents. Questions about the experience of pharmacy customers with medicines-related services could be answered on a two-point scale (yes/no) and about expectations on a three-point scale (yes/no/maybe). The main domains of the instrument were:

1. health status and regularly used medicinal products;
2. reasons for visiting pharmacy;
3. received and expected information about medicines’ use;
4. the experience and expectations of medicines-related services;
5. privacy and waiting time prior to and time of counseling.

A copy of the survey instruments can be obtained by contacting the corresponding author.

### 2.4. Data Analysis

Based on the collected data, a database was created in MS Excel. A qualitative content analysis was performed on the two open-ended questions and descriptive statistics were calculated on the multiple-choice questions. Subsequently, data was imported into a Statistical Package for Social Sciences (SPSS^®^, IBM Corporation, Armonk, NY, USA), v. 27. To compare the multiple-choice question replies of non-minorities (Estonians) and minorities (Russians), the Independent *t*-Test was used, after checking the sample homogeneity of variances with the Levene’s test. The associations of demographic characteristics were evaluated by using the Pearson Chi-Square test. The statistical significance level was set at *p* < 0.05.

In this article, Estonian-speaking and Russian-speaking study participants are presented as Estonians and Russians, respectively, due to the brevity of these concepts.

## 3. Results

### 3.1. Demographic Characteristics

The study involved 552 pharmacy customers: 323 Estonians and 229 Russians. The demographics of the two samples differed in two characteristics: the place of residence and self-evaluated health. More Russians lived in towns and were more concerned about their health than Estonians (Table 1).

According to the general sample, the use of a high number of medicines was related to lower self-assessment in health (*p* < 0.001) and the level of education and income increased proportionally (*p* < 0.001). Described results can be considered as important social aspects of medication behavior.

### 3.2. Aspects of Intercourse about Medicines at a Community Pharmacy

In the general sample, the frequency of pharmacy visits about medicines and minor illnesses was mostly indicated as sometimes: 68.8% and 64.8%, respectively. The alternative ways for study participants to solve medicines-related problems were to consult a general practitioner or trust their own knowledge. With the increase of age and number of used medicines, more people asked information about medicines (*p* < 0.001) and minor health problems (*p* < 0.003) in pharmacies.

The reasons to use medicines-related services in a pharmacy was mostly affected by the belief that the pharmacist is competent to help (78.3%). A pharmacist’s personal willingness and sufficient time to communicate about the medicines were relevant for half of the participants (53.4%). Additionally, privacy, waiting and counseling time in a community pharmacy were considered important by the participants. Most of the customers (75.7%) would be happy to wait to talk to the pharmacist for 5–10 min, whereas elderly customers would spend even more time waiting (*p* < 0.001) and Russian customers would spend more time talking to the pharmacist than Estonians (*p* < 0.001). More than half (55.2%) of the participants would prefer to have more privacy in a pharmacy when talking to the pharmacist.

Regarding medicines, questions asked in a pharmacy also differed by respondents. The concerns about medicines were more common among Russians than Estonians (*p* = 0.037), nevertheless, they sought less contact to consult a pharmacist than Estonians (*p* < 0.001). Russians also emphasized the need to know more about the used medicines (*p* = 0.002), whereas Estonians considered it more important for a pharmacist’s initiative to ask about medicines (*p* < 0.001) and to solve problems related with used medicines (*p* < 0.001). Despite the need for additional drug information and supportive advice, Russian respondents were less confident about the pharmacist’s ability to help: “Pharmacist is not a prescriber and thus unable to resolve the medicines-related problems”.

### 3.3. Pharmacist as a Mentor

Pharmacists have been mostly pictured as a trustworthy source about medicines. However, the experiences receiving medicines-related advice from pharmacists were different for Estonians and Russians in all the asked aspects (*p* < 0.001) (Table 2). Perhaps one reason for the differences is related to Estonians demonstrating a greater need for guidance by a pharmacist on how to use medicines in comparison to Russians (*p* = 0.044). Moreover, significantly less Russians indicated the need to be encouraged by a pharmacist to take the medicines as prescribed by a doctor (*p* = 0.009). The differences described may also be related to the language barrier. 

**Figure 1:**
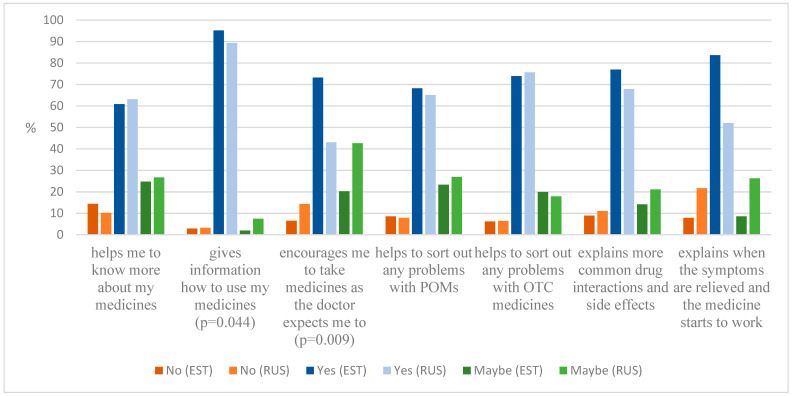
Pharmacy customers’ expectations towards the pharmacist on obtaining advice about medicines based on their mother tongue (Russian and Estonian).

Almost ¾ of all respondents would expect a pharmacist to help solve their problems related to OTC medicines’ use and to explain the most common adverse drug reactions and interactions. There were no significant differences between the expectations of Estonian and Russian customers regarding information on medicines, which indicates a similar need for consultation, but probably in different ways to receive it or the ability to ask for it (Figure 1).

Some study participants commented how their perception about pharmacists changed after learning more about what type of medicines-related information they can receive from this source. Mostly, the competence and knowledge of pharmacists were outlined: “I appreciate the pharmacist’s awareness and competence to explain the effect of the medicines and active substances. Good advice helps to cure the disease” (an Estonian respondent). A few study participants brought out the comparison with doctors: “In the media, there is no information that the pharmacist is as competent as GP” (a Russian respondent). “A pharmacist is no less competent in matters of medicines than a doctor. They can supplement the information given by the doctor” (an Estonian respondent).

### 3.4. Future Perspectives about Medicines-Related Services

Of the four medicines-related services asked, the expectations of Estonian pharmacy customers were higher than that of Russians in the case of three services. Despite the difference, both groups wanted to have advice about new medicines in the future. There was also a great interest towards the medication review service, despite the fact that there is still no practical experience of this service in Estonia (Table 3).

The results also showed that Russian customers were more confident in meeting their expectations for counseling regarding specific medicines (e.g., adverse drug reactions) and were vague about the pharmacy services in general.

## 4. Discussion

To the best of the authors’ knowledge, this is the first study evaluating how social and cultural determinants influence the use of and expectations towards medicines-related services at community pharmacies in Estonia. Following socio-cultural theory, human intellectual ability is a social process driven by intercourse with others and cultural circumstances [43]. This study indicated that the pharmacy is used as a source of information on medicines, less by Russians and more by Estonians. It may be related to the lack of actual experiences or quality of the received services and the language barrier, as well as cultural beliefs, where the doctor’s advice is more respected [14]. In addition, the organization of healthcare can also be an influence, but it should affect all ethnic groups. Recent healthcare reform in Estonia supports the GP’s central role in primary care of patients [33]. Community pharmacies are not well integrated into primary healthcare, and are sometimes described as a business entity rather than a healthcare setting [32].

Study participants gave a true picture of the Estonian and Russian pharmacy customers’ profile. Most of them were women with often up to four or even more medicines to use, and the questions and concerns were mostly related to ensure the safe and effective use of medicines. With increase of age and polypharmacy, these problems became more common. In addition, the income of the Russian survey participants was somewhat lower, thus there may be more price-sensitive patients in this population group. At the same time, access to pharmacy services was more convenient for Russian respondents, as most of them lived in larger cities where pharmacies are densely located. Nevertheless, the study results demonstrated less common experience of received medicines-related counseling at community pharmacy among Russian than Estonian respondents.

The lack of actual experiences affects the expectations of the pharmacy-based services. Based on previous research, pharmacy customers prefer convenience: location, long opening hours, etc., rather the quality of the services provided [44]. In this study, only half of the Estonian and a third of the Russian participants had experienced pharmacist consultation towards solving problems of medication use. It may refer to the dispensing of medicines rather than consulting patients on various aspects of medicines. The development of a person-centered service, in turn, can be hampered by the non-standardized service quality and/or poor integration of pharmacy services to healthcare.

In general, the interest to have other medicines-related services at a community pharmacy in the future was moderate, except service regarding new medicines. The latter clearly reflects the need for comprehensive advice to first-time medicine users, which may not be possible when counseling over the counter. Poor patient understanding about the use of medicines, or the benefits and risks of a prescribed medicine serve as a barrier to treatment outcomes and medication adherence [45]; whereby Lubi et al. showed that Estonian minorities are slightly more adhered to medicines because they have higher trust in medical authorities, their decisions and recommendations [46]. Nevertheless, in order to reach the full potential of community pharmacists, there is an urgent need to promote the role of pharmacists and pharmacies to build trustworthy relationships with patients, to better support patient use of medicines. The current study indicated that actual knowledge about pharmacists´ professional competence was insufficient in all ethnic groups. Other studies have shown that the absence of the patient–pharmacist relationship causes patients to be uncertain of the pharmacist’s role, but also that a pharmacist’s own professional identity and associated role conflict could be seen as a barrier for delivering person-centered care [47,48,49]. Therefore, a person-centered approach recognizing a patient´s preferences, values and needs to increase their independence and autonomy should be considered in the future. Advancing the patient experience regarding use of medicines has the ability to affect their decisions about treatment and, hence, health outcomes [50]. Additionally, trust towards healthcare professionals will increase with higher satisfaction of the treatment and improved health outcomes [51].

Effective communication is a prerequisite for quality and person-centered care. Language barriers pose challenges in terms of ensuring quality of care and patient safety [52,53]. The 2011 census showed that the proficiency in the Estonian language among the Russian-speaking population is a reflection of the proficiency in the Russian language among Estonians. In other words, the younger generation of Estonians is just as likely not to speak Russian as the older generation of Russians Estonian [54]. To limit the Russian language barrier among younger pharmacists in Estonia, Tallinn Health Care College started a course for pharmacists: “Working Russian language for Pharmacists” to ensure that pharmacists are able to give instructions in Russian on the use, storage, (potential) adverse drug reactions of medicines, etc. [55]. A Danish study highlighted that involving pharmacists with ethnic minority backgrounds in encounters with ethnic minorities improves access and quality of care [56]. Alternatively, implementing online translation tools, e.g., Google Translate and MediBabble may also enhance the quality of care and satisfaction among patients as well as among healthcare providers [52].

The problem of language barriers may not only be present for pharmacy customers but also for pharmacists. Recent study showed that Estonian language proficiency among Russian-speaking pharmacists was limited and was mainly influenced by daily practice, country of origin, place of residence and multiculturalism [57]. Limited knowledge of local language hampers implementing local pharmacy practice guidelines and thus does not lead to changes in the quality of the services provided. For example, community pharmacy service quality guidelines, developed in 2012, were available also in Russian only since 2021 [58].

The pharmacy environment also influences experiences about received care. More than half of the study participants outlined the importance of privacy. Lack of privacy affects the behavior of the patient during medicine consultations and can cause a patient’s limited involvement in the care decisions [59,60]. There is a greater need to have a more proactive approach towards pharmacy layouts and a system to better respond to privacy challenges [60]. Only 16% of community pharmacies in Estonia have private consultation rooms [40], but privacy is also needed in the sales area when counseling on prescription and over-the-counter medicines.

## 5. Conclusions

Experiences and expectations regarding medicines-related community pharmacy services varied across different socio-demographic and cultural population groups in Estonia. Despite the fact that Russian pharmacy customers were concerned about the use of medicines, they turned to pharmacies less than Estonians to solve these problems. It may be related to the language barrier and/or the high level of trust in the GP. These aspects need to be clarified in future research. One of the key solutions to improving the quality of healthcare for minorities is to reduce the language barrier and to increase the recognition of cultural traditions by health professionals.

## Figures and Tables

**Table 1:** Demographic characteristics and health status of pharmacy customers based on their mother tongue.

Characteristics		Estonians	Russians	Total	*p* Value
		n (%)	n (%)	N (%)	
**Gender**	Female	244 (75.8)	189 (82.5)	433 (78.6)	*p* = 0.52
	Male	78 (24.2)	40 (17.5)	118 (21.4)	
**Age**	18–34	65 (20.1)	60 (26.2)	125 (22.6)	*p* = 0.554
	35–44	73 (22.6)	41 (17.9)	114 (20.7)	
	45–54	88 (27.2)	35 (15.3)	123 (22.3)	
	55–64	28 (8.7)	25 (10.9)	53 (9.6)	
	>65	69 (21.4)	68 (29.7)	137 (24.8)	
**Education**	Primary school	1 (0.3)	1 (0.4)	2 (0.4)	*p* = 0.128
	Basic school	10 (3.1)	7 (3.1)	17 (3.1)	
	High school	111 (34.5)	69 (30.1)	180 (32.6)	
	Vocational school	83 (25.6)	49 (21.4)	132 (23.9)	
	Higher education	118 (36.5)	103 (45.0)	221 (40.0)	
**Working status**	Studying	15 (4.7)	13 (5.7)	28 (5.1)	*p* = 0.874
	Working	221 (68.6)	134 (58.5)	355 (64.4)	
	Retired pensioner	71 (22.0)	67 (29.3)	138 (25.0)	
	Disability pensioner	1 (0.3)	4 (1.7)	5 (0.9)	
	Not working	6 (1.9)	1 (0.4)	7 (1.3)	
	Other	8 (2.5)	10 (4.4)	18 (3.3)	
**Place of residence**	Rural area	33 (10.3)	5 (2.2)	38 (6.9)	*p* < 0.001
	Small town	51 (15.9)	4 (1.7)	55 (10.0)	
	Town, <50,000 inhabitants	117 (36.6)	63 (27.5)	180 (32.8)	
	Town, >50,000 inhabitants	119 (37.2)	157 (68.6)	276 (50.3)	
**Income**	<EUR 500	59 (18.4)	59 (26.5)	118 (21.7)	*p* = 0.79
	EUR 501–1000	151 (47.0)	98 (43.9)	249 (45.8)	
	EUR 1001–2000	103 (32.1)	58 (26.0)	161 (29.6)	
	>EUR 2000	8 (2.5)	8 (3.6)	16 (2.9)	
**General health status**	Very poor	1 (0.3)	2 (0.9)	3 (0.5)	*p* = 0.006
	Poor	21 (6.5)	18 (7.9)	39 (7.2)	
	Fair	108 (33.4)	97 (42.3)	205 (37.1)	
	Good	142 (44.0)	90 (39.3)	232 (42.0)	
	Very good	51 (15.8)	22 (9.6)	73 (13.2)	
**Regularly taken medicines**	No	137 (42.5)	80 (34.9)	217 (39.3)	*p* = 0.141
	Yes, <4	130 (40.2)	107 (46.8)	237 (42.9)	
	Yes, 5–8	43 (13.3)	28 (12.2)	71 (12.9)	
	Yes, >8	13 (4.0)	14 (6.1)	27 (4.9)	

**Table 2:** Experiences of pharmacy customers on medicines-related counseling based on their mother tongue (Russian and Estonian).

			Levene’s Test for Equality of Variances				*t*-Test for Equality of Means				
Have You Experienced That the Pharmacist…	Mother Tongue	Mean (Scale Range 0–1) ± SD	F	*p* Value	t	df	*p* Value (2-Tailed)	Mean Difference	Std. Error Diffe-rence	95% Confidence Interval of the Difference	
										Lower	Upper
Helped me to know more about my medicines	Estonian	0.59 ± 0.49	0.42	0.516	3.7	546	<0.001	0.16	0.04	0.07	0.24
	Russian	0.43 ± 0.50									
Helped me to understand better how to use my medicines	Estonian	0.94 ± 0.24	781.05	<0.001	13.04	301.3	<0.001	0.46	0.04	0.4	0.53
	Russian	0.48 ± 0.50									
Encouraged me to take my medicines as the doctor expects me to	Estonian	0.7 ± 0.46	5.30	0.022	11.36	504.63	<0.001	0.44	0.04	0.36	0.52
	Russian	0.26 ± 0.44									
Helped me to sort out any problems with my prescription medicines	Estonian	0.45 ± 0.50	63.15	<0.001	4.17	518.07	<0.001	0.17	0.04	0.09	0.25
	Russian	0.28 ± 0.45									
Helped me to sort out any problems with my OTC medicines	Estonian	0.55 ± 0.50	13.03	<0.001	3.4	498.99	<0.001	0.17	0.04	0.09	0.25
	Russian	0.38 ± 0.48									
Talked about the medications you are taking regarding more common side-effects and interactions	Estonian	0.51 ± 0.50	52.97	<0.001	4.84	512.23	<0.001	0.2	0.04	0.12	0.28
	Russian	0.31 ± 0.46									
Talked about when the symptoms of the disease are relieved and the medicine starts to work	Estonian	0.65 ± 0.48	43.26	<0.001	10.94	524.29	<0.001	0.42	0.04	0.35	0.45
	Russian	0.23 ± 0.42									

**Table 3:** Expectations of pharmacy customers on medicines-related services based on their mother tongue (Estonian and Russian).

				Levene’s Test for Equality of Variances			*t*-Test for Equality of Means				
Would You Like to Have in the Future?	Mother Tongue	Mean (Scale Range 0–2) ± SD	F	*p* Value	t	df	*p* Value (2-Tailed)	Mean Difference	Std. Error Difference	95% Confidence Interval of the Difference	
										Lower	Upper
Advice about using medicines	Estonian	1.64 ± 0.54	30.28	<0.001	4.55	421.4	<0.001	0.25	0.05	0.14	0.35
	Russian	1.39 ± 0.68									
Advice about new medicines	Estonian	1.81 ± 0.43	52.27	<0.001	3.42	375.64	<0.001	0.16	0.05	0.07	0.26
	Russian	1.65 ± 0.63									
Discussion about medicines	Estonian	1.41 ± 0.74	11.36	<0.001	−2.4	522.76	0.017	−0.14	0.06	−0.26	−0.03
	Russian	1.55 ± 0.64									
Review of your medicines use	Estonian	1.44 ± 0.69	3.28	0.071	0.76	541	0.445	0.05	0.06	−0.07	0.17
	Russian	1.39 ± 0.75									

## Data Availability

Not applicable.
